# Determinants of Health-Related Quality of Life (HRQoL) in the Multiethnic Singapore Population – A National Cohort Study

**DOI:** 10.1371/journal.pone.0067138

**Published:** 2013-06-27

**Authors:** Melvin Khee-Shing Leow, Konstadina Griva, Robin Choo, Hwee-Lin Wee, Julian Thumboo, E. Shyong Tai, Stanton Newman

**Affiliations:** 1 Department of Endocrinology, Tan Tock Seng Hospital, Singapore, Republic of Singapore; 2 Singapore Institute for Clinical Sciences, A*STAR, Singapore, Republic of Singapore; 3 Yong Loo Lin School of Medicine, National University of Singapore, Singapore, Republic of Singapore; 4 Office of Clinical Sciences, Duke-NUS Graduate School of Medicine, Singapore, Republic of Singapore; 5 Department of Psychology, Faculty of Science, National University of Singapore, Singapore, Republic of Singapore; 6 Department of Rheumatology & Immunology, Singapore General Hospital, Singapore, Republic of Singapore; 7 Department of Pharmacy, Faculty of Science, National University of Singapore, Singapore, Republic of Singapore; 8 Yong Loo Lin School of Medicine, National University of Singapore, Singapore, Republic of Singapore; 9 Division of Endocrinology, Department of Medicine, National University of Singapore, Singapore, Republic of Singapore; 10 School of Community & Health Sciences, City University, London, United Kingdom; CUNY, United States of America

## Abstract

**Background:**

HRQoL is an important outcome to guide and promote healthcare. Clinical and socioeconomic factors may influence HRQoL according to ethnicity.

**Methodology:**

A multiethnic cross-sectional national cohort (N = 7198) of the Singapore general population consisting of Chinese (N = 4873), Malay (N = 1167) and Indian (N = 1158) adults were evaluated using measures of HRQoL (SF-36 version 2), family functioning, health behaviours and clinical/laboratory assessments. Multiple regression analyses were performed to identify determinants of physical and mental HRQoL in the overall population and their potential differential effects by ethnicity. No a priori hypotheses were formulated so all interaction effects were explored.

**Principal Findings:**

HRQoL levels differed between ethnic groups. Chinese respondents had higher physical HRQoL (PCS) than Indian and Malay participants (p<0.001) whereas mental HRQoL (MCS) was higher in Malay relative to Chinese participants (p<0.001). Regressions models explained 17.1% and 14.6% of variance in PCS and MCS respectively with comorbid burden, income and employment being associated with lower HRQoL. Age and family were associated only with MCS. The effects of gender, stroke and musculoskeletal conditions on PCS varied by ethnicity, suggesting non-uniform patterns of association for Chinese, Malay and Indian individuals.

**Conclusions:**

Differences in HRQoL levels and determinants of HRQoL among ethnic groups underscore the need to better or differentially target population segments to promote well-being. More work is needed to explore HRQoL and wellness in relation to ethnicity.

## Introduction

Health-related quality of life (HRQoL) is an important outcome used in a wide variety of medical research to ascertain aspects of well-being in settings of health and disease. HRQoL focuses on the aspects of an individual’s life that is impinged on by health, disease and its treatment, and is a fundamental component of successful ageing covering life expectancy, life satisfaction, mental and psychological health, physical health and functioning. This is especially relevant to countries such as Singapore having populations that are rapidly ageing and at risk of age-related diseases that can affect the nation as a whole with respect to productivity and consumption of health care resources. In 2011, 9.3% of the total population of Singapore were aged 65 years and above, as compared with 3.4% in 1970 [1].

There is a growing recognition of the importance of HRQol, as evidenced by the US National Institutes of Health PROMIS initiative, UK Government guidance and a burgeoning number of reports in leading journals [2]–[7]. Biomedical/clinician-assessed measures of health status such as comorbidity are often unable to capture individuals’ perspective and often correlate poorly with patient-reported outcomes such as HRQoL and/or subjective function [8]. For instance, the DOPPS study has shown that at higher levels of comorbidity, African American patients on dialysis report better physical wellbeing (as measured by Short Form Survey-36) than non-African Americans [9]. Scales such as the SF-36 provide a measure of patients’ perceived states, capabilities and functioning in physical, psychological and social areas of life that complement objective clinical markers and disease indicators. They have been shown to predict morbidity and subsequent mortality in community samples even after adjusting for objective measures of risk and comorbidity [10]–[12]. HRQoL hence provides a measure that is sensitive to the patients’ perspective and subjective experience of health and illness that expand upon clinical measures. This clearly supports the consideration of HRQoL outcomes in planning, implementation and evaluation of health provision and policies on a wider level. Pertinent to this is the documentation of HRQoL outcomes and the identification of factors that may be associated with these outcomes across general populations and different population segments.

The role of socioeconomic factors and comorbidities on HRQoL is well recognized. What is less well understood is the possible impact of specific biochemical and metabolic parameters or biological measures such as body weight and blood pressure on HRQoL. Although such markers denote poor health and/or increased risk for poor health and infirmity, they have not shown consistent associations with HRQoL [13], [14].

Similarly, despite well-established evidence demonstrating ethnic differences and racial disparities in health outcomes [15], the relationship between ethnicity and HRQoL is limited and fraught with inconsistencies, thereby hindering any guidance towards targeted public health programs. Studies on community adult samples and patients with diabetes indicate worse physical and mental HRQoL for Indian respondents [16]–[18], whereas in a study on healthy adolescents, Indian respondents fared best on overall and emotional quality of life [16] than other ethnic groups. Notably, the observed HRQoL differences persist even after adjustment for sociodemographic parameters [17], [18], indicating that they do not simply reflect casemix differences. None of the previous work have examined if ethnic differences persist when specific biochemical and biological parameters are taken into account. Clearly more sufficiently powered population-based studies are warranted to elucidate ethnic variations in HRQoL and identify drivers of HRQoL especially in the context of different population segments and ethnicities.

There are important ethnic variations in clinical risk factors, biochemical markers, health behaviors and certain sociocultural parameters that may differentially affect HRQoL. A case in point relates to differences in waist-hip ratio being highest in Indians, intermediate in Malays and lowest in Chinese, in parallel with insulin resistance and high-sensitivity C-reactive protein (hs-CRP) [19]–[22]. Differences in other cardiovascular risk factors (i.e. obesity, cholesterol, diabetes) between the three ethnic groups in Singapore (higher mainly in Indian and Malay relative to Chinese counterparts) have also been noted [23]. Indices of socioeconomic status (i.e. education, English or multi-language literacy, income) on the other hand are higher in Indian than Chinese [24]. Variation in health practices and behaviors such as smoking, diet or physical activity may underpin these clinical differences and further impact upon HRQoL. For instance, national health surveillance data have shown that smoking rates are higher amongst Malays compared to Chinese and Indians [25] whereas rates of regular exercise are substantially lower in Chinese compared with the other two ethnic groups [23]. While the recognition of differential risk profiles in population subgroups have led to a more focused approach for the national health promotion program (e.g. National Healthy Lifestyle Programme (NHLP)), much remains unknown about ethnicity on well-being and wellness outcomes, and the contribution of clinical variables on HRQoL.

Documenting HRQoL and determinants across races would add valuable information on wellness of a nation, complement risk/disease profiling and may allow greater customization of health promotion programs.

The aims of this present study are as follows:

- To document and compare HRQoL levels in the three main ethnic groups in Singapore: Chinese, Malay, Indian
- To identify determinants of the physical and mental HRQoL in a multiethnic population
- To explore if determinants of HRQoL differ in these three ethnic groups.

We hypothesize that comorbid burden, lower socioeconomic standing and family functioning are associated with lower HRQoL. No *a priori* hypotheses were formulated with respect to biomarkers or ethnic differences on HRQoL or its determinants as previous evidence is either lacking or inconsistent.

## Methods

### Study Design and Cohort

**Figure 1:**
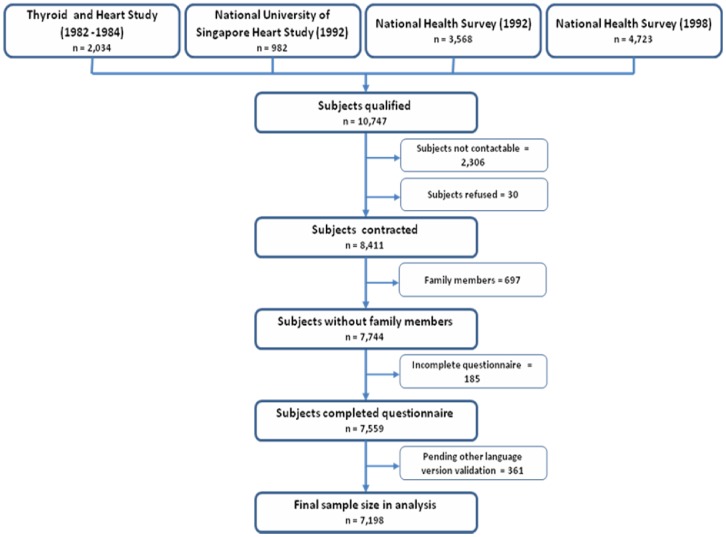
Inclusion of subjects for study of HRQoL.

The Singapore Prospective Study Program (SP2) is a national population-based cross-sectional study inclusive of N = 7198 community-dwelling individuals between the ages of 21 to 95 years, selected by disproportionate stratified sampling to ensure representation of the ethnic composition of Singapore population: Chinese, Malay and Indians. The overall SP2 cohort consists of samples from four related epidemiological studies (Thyroid and Heart Study, the National Health Survey (1992), the National University of Singapore Heart Study (1993–1995) and the National Health Survey (1998) (Figure 1). Although their clinical objectives were different (i.e. screening and monitoring for particular conditions), all four epidemiological surveys had common data collection instruments and comparable clinical assessment protocols to allow merging of datasets. Recruitment of the original four epidemiological cohorts occurred between 1992–1998, whereas data on HRQoL that formed the SP2 dataset were collected between 2003–2007 as part of the scheduled follow-up assessments. There were no significant effect of original cohort source nor any between-study effects related to year of collection on reported outcomes.

### Ethics

Ethical approval was obtained from local Institutional Review Board (SingHealth IRB) and written informed consent was obtained from all participants.

### Measures, Instruments and Procedure

Each subject underwent a physical examination including a blood sample for fasting laboratory evaluation and completed interviewer-administered questionnaires. The sociodemographic questionnaire included data on age, gender, ethnicity (Chinese, Malay, or Asian-Indian) education (no education, primary, secondary and above), monthly income (<$2000, $2000–3999, $4000–5999, $6000–9999, > = $10,000), smoking (current smoker/noncurrent or nonsmoker), alcohol consumption (current drinker and noncurrent drinker) and physical activity measured with International Physical Activity Questionnaire (IPAQ) [26], [27]. Information on chronic diseases and relevant medication use was captured in the questionnaire assessment [14].

The Short Form health survey with 36 questions (SF36 version 2) [28], [29]is a well-documented scoring system that has been widely used and validated as a HRQoL assessment tool for the general population as well as patient groups [30]–[33]. It includes 8 subscales to evaluate individuals’ perceptions of their health and the impact of their health on physical, social emotional domains/functioning: Physical Functioning, Physical Role (i.e., role limitations caused by physical problems), Pain, General Health, Vitality, Social Functioning, Emotional Role (i.e., role limitations caused by emotional problems), and Mental Health. These 8 subscales are combined into Physical Component Score (PCS) and a Mental Component Score (MCS) [28] which are the foci of this paper. Scores range from 0 to 100, with higher scores indicating better HRQoL for each domain. Its psychometrics properties have been established in several countries [34], [35].

The 3 item Family Functioning Measure (FFM) was used to assess the quality of interactions among family members [36]. It has previously been validated in a study in Singapore [37].

### Metabolic Phenotyping of the Study Population

Anthropometric indices [body weight (kilograms); standing height (meters); waist and hip circumferences] were measured with the subject wearing light clothing and no shoes by trained research nurses. Waist circumference was taken as the narrowest measurement midway between the xiphoid sternum and umbilicus, while the hip circumference was taken as the widest measurement at the level of the greater trochanters. Sitting blood pressure (BP) was measured after the subject was at rest for 5 min using an automated sphygmomanometer. Baseline blood samples were drawn after an overnight fast and taken for measurements of plasma glucose, lipid profile, insulin, high sensitivity C-reactive protein (hs-CRP), among a series of other laboratory investigations meant for other studies. Homeostasis model assessment-insulin resistance index (HOMA-IR) was calculated using the HOMA-IR formula, defined as fasting insulin (mU/L) x fasting plasma glucose (mmol/L)/22.5. This equation provides a convenient estimate of insulin resistance that has been validated by comparison with results of glucose clamp studies [38].

### Laboratory Biochemical Assays

Blood was sampled in the morning by venipuncture after an overnight fast of at least 8 hours and transferred on ice immediately and centrifuged at 2500 rpm for 15 min, and the supernatant separated and stored at −20°C until analysis. The analytical performance of these methods was within the specifications of the analyzers.

### Statistical Analysis

**Figure 2:**
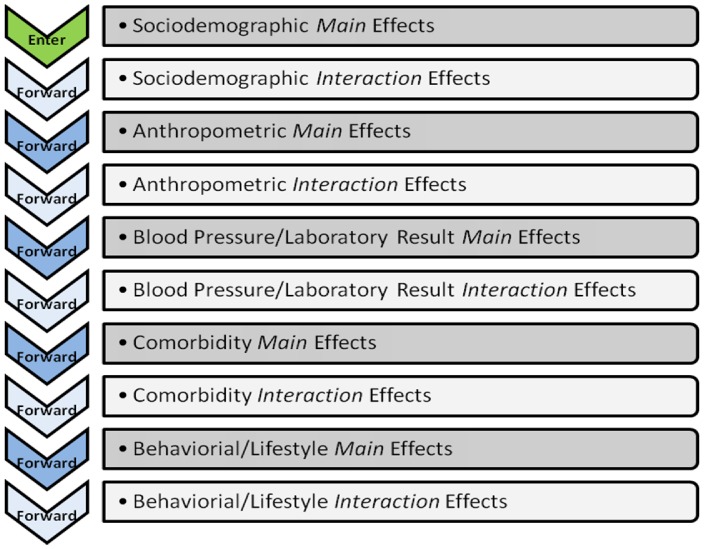
Hierarchical Forward Regression for Main and Interaction Effects.

Statistical analyses were performed using STATA version 10.0; StataCorp, Texas, USA) except for hierarchical regressions that were run with SPSS (version 16.0; SPSS, Inc., Chicago, IL). Differences in sociodemographic, clinical/laboratory measures between ethnic groups were assessed with ANOVAs (for continuous variables) or chi-square test (categorical variables). The associations with HRQoL (PCS; MCS) of demographic variables (including ethnicity), sociodemographic and clinical/laboratory variables were evaluated using univariate tests and hierarchical regressions. Variance inflation factors (VIF), a post-estimate method was used to check that no multi-collinearity exists before running the hierarchical analyses. Hierarchical regression models were constructed by sequentially adding predictors into five blocks: (i) sociodemographics factors (age, marital status, education, ethnicity, employment, income, housing) (enter method), (ii) anthropometric measurements (forward selection), (iii) systolic/diastolic blood pressure and laboratory data (forward selection), (iv) clinical variables (i.e. comorbidities) and medication (forward selection), (v) other behavioral variables (smoking/alcohol consumption, family function measure and physical activity expenditure) (forward selection). To explore ethnic variations in HRQoL determinants, interaction terms were forward selected after each respective block of the main effects. For instance, interaction terms of sociodemographic variables by ethnicity were forward selected after its main effects were entered; next, the interaction terms of the anthropometric parameters by ethnicity were forward selected after its main effects as well. This applies to the interaction effects of laboratory variables, comorbidities and other behavioral variables (Figure 2). Chinese were used as the reference group for each interaction term.

All analyses were completed on complete cases (no missing data imputation). Missing data for income and some anthropometric/laboratory variables were over 30%, hence imputation procedures were not deemed suitable.

## Results

### Characteristics of the Study Cohort

The baseline characteristics of the total study cohort and the three ethnic subgroups are shown in Table 1 (sociodemographic profile) and Table 2 (clinical and lifestyle profile). There were significant differences (*p*<0.001) in sociodemographic factors (except gender), anthropometric measurements, systolic/diastolic blood pressure and laboratory data, clinical variables (i.e. comorbidities) and medication, and other behavioral variables (smoking/alcohol consumption, FFM and physical activity expenditure) among the ethnic groups. Details pertaining to the ANOVA post-hoc analysis can be found in Table S1. The general pattern indicated that the Chinese respondents had ‘healthier’ clinical profile than Malay and Indian participants (e.g. lower body mass index, blood pressure, diabetes prevalence, lower fasting glucose, low density lipoprotein cholesterol (LDL-C), insulin resistance and high sensitivity C-reactive protein (hs-CRP)), which probably underlie the well known differences in cardio-metabolic risks between the 3 main ethnicities in this country (See Table S1 for post hoc analyses). The socioeconomic background of Malay respondents as indexed by education, housing type, employment and income was lower than that of Chinese and Indian subgroups.

**Table 1:** Socio-demographics of study participants.

Variables	*Miss* *(%)*	Overalln = 7,198	Chinesen = 4,873 (67.70%)	Malayn = 1,167 (16.21%)	Indiann = 1,158 (16.09%)	P-value
SF-36 norm-based scores, *mean (SD)*						
Physical component summary score	0.00%	50.00 (9.82)	50.91 (8.98)	49.12 (10.61)	47.06 (11.61)	<0.001†
Mental component summary score	0.00%	49.55 (10.20)	49.22 (10.22)	50.91 (9.68)	49.59 (10.53)	<0.001†
Age (years)						
Mean (SD)	0.00%	49.4 (12.58)	49.47 (12.69)	47.78 (12.44)	50.69 (12.07)	<0.001†
Gender, *n (%)*	0.00%					0.365
Male		3,416 (47.46)	2,294 (47.08)	576 (49.36)	546 (47.15)	
Female		3,782 (52.54)	2,579 (52.92)	591 (50.64)	612 (52.85)	
Marital status, *n (%)*	0.00%					<0.001‡
Currently married		5,639 (78.34)	3,771 (77.39)	934 (80.03)	934 (80.66)	
Never married		984 (13.67)	773 (15.86)	111 (9.51)	100 (8.64)	
Separated/divorced		195 (2.71)	119 (2.44)	39 (3.34)	37 (3.20)	
Widowed		380 (5.28)	210 (4.31)	83 (7.11)	87 (7.51)	
Education level, *n (%)*	0.00%					<0.001‡
Primary		1,940 (26.95)	1,328 (27.25)	306 (26.22)	306 (26.42)	
Secondary		2,567 (35.66)	1,567 (32.16)	554 (47.47)	446 (38.51)	
Tertiary		2,691 (37.39)	1,978 (40.59)	307 (26.31)	406 (35.06)	
Employment status, *n (%)*	0.00%					<0.001‡
Working		4,893 (67.98)	3,367 (69.1)	783 (67.10)	743 (64.16)	
Student		36 (0.5)	23 (0.47)	7 (0.60)	6 (0.52)	
Housewife		1393 (19.35)	854 (17.53)	274 (23.48)	265 (22.88)	
Retired		651 (9.04)	471 (9.67)	78 (6.68)	102 (8.81)	
Unemployed (able)		120 (1.67)	86 (1.76)	13 (1.11)	21 (1.81)	
Unemployed (unable)		49 (0.68)	34 (0.70)	6 (0.51)	9 (0.78)	
Others		56 (0.78)	38 (0.78)	6 (0.51)	12 (1.04)	
Income category, *n (%)*	42.36%					<0.001‡
< $2000		1,219 (29.38)	664 (24.17)	290 (42.40)	265 (36.91)	
$2000–$3999		1,378 (33.21)	857 (31.20)	277 (40.50)	244 (33.98)	
$4000–$5999		702 (16.92)	523 (19.04)	79 (11.55)	100 (13.93)	
$6000–$9999		540 (13.02)	432 (15.73)	29 (4.24)	79 (11.00)	
>$10,000		310 (7.47)	271 (9.87)	9 (1.32)	30 (4.18)	
Housing type, *n (%)*	0.00%					<0.001‡
Small public housing		1,267 (17.6)	874 (17.94)	202 (17.31)	191 (16.49)	
Large public housing		4,797 (66.64)	3,059 (62.77)	909 (77.89)	829 (71.59)	
Private housing		1,134 (15.75)	940 (19.29)	56 (4.80)	138 (11.92)	

† ANOVA F statistics are significant at 5% level.

‡ Pearson Chi^2^ statistics are significant at 5% level.

**Table 2:** Clinical and lifestyle profile of study participants.

Variables	*Miss* *(%)*	Overalln = 7,198	Chinesen = 4,873 (67.70%)	Malayn = 1,167 (16.21%)	Indiann = 1,158 (16.09%)	P-value
Anthropometric measurements, *mean (SD)*						
Height (cm)	33.63%	163.29 (8.92)	163.6 0(8.63)	162.13 (9.22)	162.94 (9.74)	<0.001†
Weight (kg)	33.63%	63.71 (12.92)	61.55 (12.12)	68.91 (13.69)	68.47 (13.02)	<0.001†
Body mass index	33.63%	23.85 (4.23)	22.91 (3.67)	26.21 (4.73)	25.81 (4.55)	<0.001†
Waist circumference (cm)	33.72%	84.15 (11.24)	81.90 (10.63)	88.22 (11.35)	90.27 (10.4)	<0.001†
Hip circumference (cm)	33.72%	98.68 (9.23)	96.97 (8.4)	102.50 (9.79)	102.72 (9.92)	<0.001†
Waist-hip ratio	33.72%	0.85 (0.08)	0.84 (0.08)	0.86 (0.08)	0.88 (0.09)	<0.001†
Blood pressure measurements, *mean (SD)*						
Systolic blood pressure (mmHg)	33.63%	132.07 (20.86)	130.79 (20.63)	136.15 (20.56)	134.01 (21.53)	<0.001†
Diastolic blood pressure (mmHg)	33.63%	77.78 (10.79)	77.20 (10.81)	79.64 (10.80)	78.63 (10.46)	<0.001†
Laboratory measurements, *mean (SD)*						
HOMA-IR	39.73%	1.95 (2.11)	1.66 (1.67)	2.21 (2.29)	2.99 (3.08)	<0.001†
log Insulin (mU/L)	33.66%	1.88 (0.66)	1.77 (0.63)	2.00 (0.62)	2.23 (0.64)	<0.001†
log Fasting plamsa glucose (mmol/L)	33.62%	1.61 (0.21)	1.59 (0.18)	1.65 (0.27)	1.69 (0.27)	<0.001†
log High-density lipoprotein (mmol/L)	33.62%	0.26 (0.26)	0.30 (0.26)	0.21 (0.24)	0.12 (0.25)	<0.001†
log Low-density lipoprotein (mmol/L)	33.62%	1.15 (0.26)	1.13 (0.26)	1.21 (0.28)	1.19 (0.25)	<0.001†
log Cholesterol (mmol/L)	33.62%	1.63 (0.18)	1.63 (0.18)	1.67 (0.19)	1.63 (0.18)	<0.001†
log Triglyceride (mmol/L)	33.62%	0.15 (0.51)	0.1 (0.52)	0.28 (0.50)	0.24 (0.46)	<0.001†
log C-reactive protein (mg/L)	35.70%	0.17 (1.20)	−0.06 (1.14)	0.51 (1.16)	0.84 (1.17)	<0.001†
History of co-morbidities, *n (%)*						
Hypertension	33.63%	1,929 (40.38)	1,285 (38.63)	307 (45.41)	337 (43.48)	0.001‡
Diabetes mellitus	29.59%	2,080 (41.04)	1,260 (36.04)	374 (50.75)	446 (53.41)	<0.001‡
Coronary heart disease	0.10%	204 (2.84)	106 (2.18)	23 (1.97)	75 (6.48)	<0.001‡
Cerebrovascular accident (stroke)	0.00%	111 (1.54)	65 (1.33)	10 (0.86)	36 (3.11)	<0.001‡
Asthma lung disease	0.00%	338 (4.70)	155 (3.18)	70 (6.00)	113 (9.76)	<0.001‡
Cancer	0.00%	65 (0.90)	54 (1.11)	4 (0.34)	7 (0.60)	0.023‡
Musculoskeletal illness	0.00%	1,491 (20.71)	1,066 (21.88)	164 (14.05)	261 (22.54)	<0.001‡
On medication (hypertensive/diabetics/lipid-lowering)	0.00%	1,844 (25.62)	1,166 (23.93)	291 (24.94)	387 (33.42)	<0.001‡
Smoking, *n (%)*	0.00%					<0.001‡
Never smoke		5,693 (79.09)	3,919 (80.42)	838 (71.81)	936 (80.83)	
Ever smoke		587 (8.16)	389 (7.98)	110 (9.43)	88 (7.60)	
Currently smoke		918 (12.75)	565 (11.59)	219 (18.77)	134 (11.57)	
Alcohol consumption, *n (%)*	0.00%					<0.001‡
Never drink		4,645 (64.53)	2,767 (56.78)	1,085 (92.97)	793 (68.48)	
Ever drink		922 (12.81)	749 (15.37)	52 (4.46)	121 (10.45)	
Currently drink		1,631 (22.66)	1,357 (27.85)	30 (2.57)	244 (21.07)	
Family function measure, *mean (SD)*	0.00%	58.69 (17.99)	57.34 (17.77)	60.87 (17.01)	62.19 (19.17)	<0.001†
Physical energy expenditure, *mean (SD)*	0.06%	0.72 (0.83)	0.67 (0.79)	0.84 (0.92)	0.81 (0.91)	<0.001†

† ANOVA F statistics are significant at 5% level.

‡ Pearson Chi^2^ statistics are significant at 5% level.

### Univariate Associations

Age, gender, ethnicity, marital status, education, income, comorbidities, anthropometric and measures indicative of worse clinical status were all significantly associated with physical and mental HRQoL in the total sample (Table S2). Clinical measures were related to MCS and yet not consistently for all variables (i.e. comorbid conditions, laboratory measures) and across all three ethnic groups. HRQoL levels differed between the three ethnic groups: PCS (F = 106.1, *p*<0.001) and MCS (F = 21.4, *p*<0.001). Chinese respondents reported the highest physical HRQoL (i.e.PCS) followed by Malay and Indian participants. All three paired mean differences in PCS were significant in the post-hoc comparisons (*p*<0.001). With respect to MCS, post-hoc comparisons showed that Malay respondents had significantly higher MCS scores than Chinese (*p*<0.001) and Indians (*p*<0.001). There was no significant mean difference in MCS between the Indian and Chinese participants.

### Multivariate Associations

Hierarchical multiple regression analyses were conducted for the total cohort with interaction terms being entered following each block of main effects. The results indicated that the main effects of being widowed, housewife or retired, higher income groups, larger waist circumference, having stroke, diabetes, coronary heart disease (CHD), asthma/lung disease, musculoskeletal conditions, previous alcohol consumption, physical activity were significant multivariate predictors, and as well as interaction terms of gender, stroke and musculoskeletal conditions by ethnicity, accounting for 17.1% of the variance in PCS (Table 3). Age and ethnicity (Chinese) significant at point of entry ceased to be significant in the final PCS model.

**Table 3:** PCS Hierarchical Regression (Final).

	OverallAdj. R^2^ = 17.14%N = 2,678		Interaction Term Malay vs. Chinese		Interaction Term Indian vs. Chinese	
Variables	β	P-value	β	P-value	β	P-value
						
*(Constant)*	*57.600*	*0.000*				
Age (years)	−0.019	0.402				
Gender						
Male *(Base reference)*						
Female	−0.583	0.253	−2.656	**0.010** *	−2.177	**0.026** *
Ethnic group						
Chinese *(Base reference)*						
Malay	0.390	0.777				
Indian	−1.091	0.381				
Marital status						
Currently married *(Base reference)*						
Never married	−0.757	0.197				
Separated/divorced	0.851	0.437				
Widowed	−2.320	**0.023** *				
Education level						
Primary *(Base reference)*						
Secondary	0.285	0.656	−0.880	0.537	−1.495	0.247
Tertiary	−0.264	0.704	−1.096	0.468	0.965	0.456
Employment status						
Working *(Base reference)*						
Student	0.009	0.997				
Housewife	−2.089	**0.000** *				
Retired	−1.893	**0.027** *				
Unemployed (able)	−1.698	0.239				
Unemployed (unable)	0.130	0.946				
Others	−8.809	**0.002** *				
Income category						
< $2000 *(Base reference)*						
$2000–$3999	0.770	0.105				
$4000–$5999	0.703	0.226				
$6000–$9999	2.279	**0.001** *				
>$10,000	3.101	**0.000** *				
Housing type						
Small public housing *(Base reference)*						
Large public housing	−0.239	0.651				
Private housing	−1.205	0.086				
Anthropometric measurements						
Waist circumference (cm)	−0.062	**0.001** *				
History of co-morbidities						
Diabetes mellitus	−1.883	**0.005** *				
Coronary heart disease	−2.808	**0.010** *				
Cerebrovascular accident (stroke)	−7.759	**0.000** *	21.599	**0.002** *	−3.162	0.429
Asthma lung disease	−2.697	**0.026** *	−2.671	0.210	3.488	0.066
Cancer	−3.713	0.057				
Musculoskeletal illness	−3.508	**0.000** *	−2.638	**0.048** *	−4.658	**0.000** *
Alcohol consumption						
Never drink *(Base reference)*						
Ever drink	1.699	**0.002** *				
Currently drink	0.574	0.193				
Physical energy expenditure per day	−0.001	**0.005** *				

* Beta coefficients are significant at 5% level.

The regression model to predict MCS in the total sample explained 14.6% of the variance with main effects of age, ethnicity, secondary education, higher income, insulin, stroke, asthma/lung diseases, musculoskeletal conditions, medication, smoking, physical energy expenditure and family functioning emerging as significant determinants, and as well as interaction terms of insulin and high density lipoprotein cholesterol by ethnicity in the final MCS model (Table 4). Further detailed results of the hierarchical multiple regression analyses are found in Table S3 and Table S4 under the supporting (supplementary) data of this paper.

**Table 4:** MCS Hierarchical Regression (Final).

	OverallAdj. R^2^ = 14.61%N = 2,678		Interaction Term Malay vs. Chinese			Interaction Term Indian vs. Chinese	
Variables	β	P-value	β	P-value		β	P-value
*(Constant)*	*35.331*	*0.000*					
Age (years)	0.187	**0.000** *					
Gender							
Male *(Base reference)*							
Female	−0.922	0.051					
Ethnic group							
Chinese *(Base reference)*							
Malay	0.850	0.638					
Indian	5.533	**0.002** *					
Marital status							
Currently married *(Base reference)*							
Never married	0.354	0.547					
Separated/divorced	0.281	0.797					
Widowed	−1.106	0.273					
Education level							
Primary *(Base reference)*							
Secondary	1.247	**0.017** *					
Tertiary	0.952	0.111					
Employment status							
Working *(Base reference)*							
Student	−3.169	0.212					
Housewife	0.596	0.309					
Retired	0.081	0.924					
Unemployed (able)	−2.509	0.081					
Unemployed (unable)	−0.031	0.987					
Others	1.222	0.655					
Income category							
< $2000 *(Base reference)*							
$2000–$3999	1.857	**0.000** *					
$4000–$5999	0.826	0.155					
$6000–$9999	1.798	**0.006** *					
>$10,000	2.748	**0.001** *					
Housing type							
Small public housing *(Base reference)*							
Large public housing	−0.746	0.158					
Private housing	−0.707	0.314					
Blood pressure measurements							
Diastolic blood pressure (mmHg)	0.025	0.172					
Laboratory measurements							
log Insulin (mU/L)	−0.762	**0.040** *	0.721	0.382		−2.023	**0.010** *
log High-density lipoprotein (mmol/L)	−1.184	0.201	1.982	0.288		−5.887	**0.001** *
History of co-morbidities							
Cerebrovascular accident (stroke)	−3.435	**0.045** *					
Asthma lung disease	−2.591	**0.002**					
Musculoskeletal illness	−3.890	**<2e-16** *					
On medication (hypertensive/diabetics/lipid-lowering)	−1.836	**0.000** *					
Smoking							
Never smoke *(Base reference)*							
Ever smoke	−1.766	**0.004** *					
Currently smoke	−1.608	**0.007** *					
Family function measure	0.087	**0.000** *					
Physical energy expenditure per day	0.000	**0.011** *					

* Beta coefficients are significant at 5% level.

**Figure 3:**
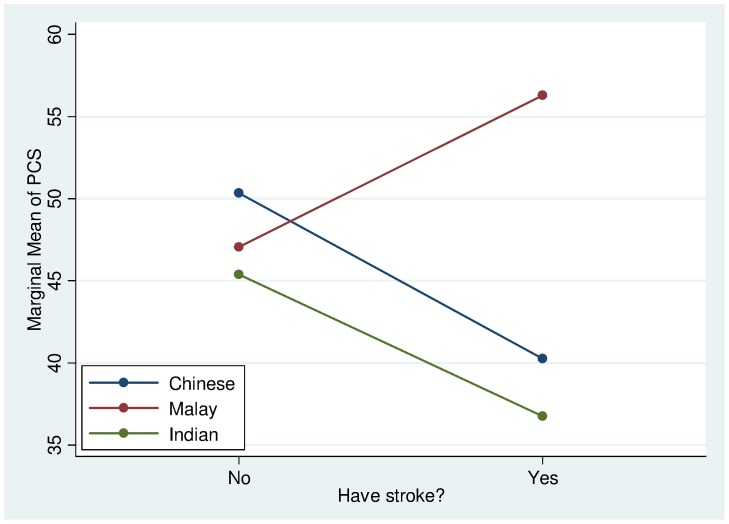
PCS Interaction plot between ethnicity and stroke.

**Figure 4:**
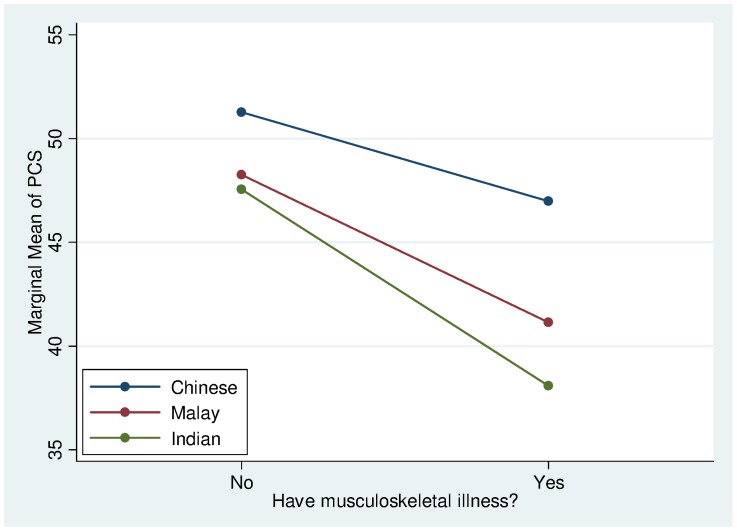
PCS Interaction plot between ethnicity and musculoskeletal illness.

**Figure 5:**
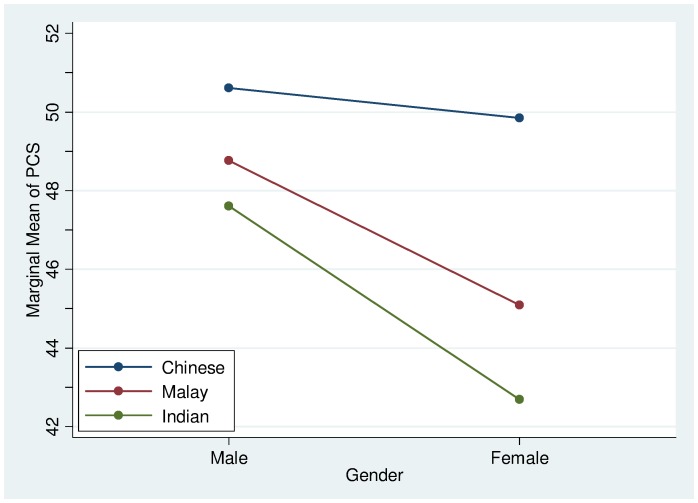
PCS Interaction plot between ethnicity and gender.

The significant interaction effects indicate that the associations of certain sociodemographic, clinical/laboratory variables to HRQoL were not uniformly observed in all ethnic groups. The effect of stroke on PCS was more severe for the Chinese and Indians but less for the Malays (Figure 3). Musculoskeletal conditions were found to have a greater adverse effect on PCS for the Malays and Indians but less so for the Chinese (Figure 4). Generally, the PCS of females is slightly lower than the males across all ethnic groups. In particular, it was found that the Malay and Indian females had a much lower PCS than their Chinese counterparts (Figure 5).

**Figure 6:**
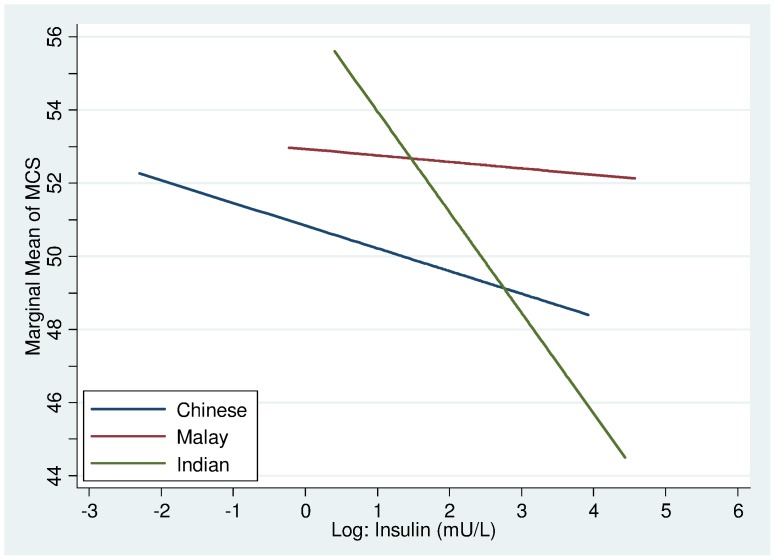
MCS Interaction plot between ethnicity and log Insulin.

In general, the serum insulin was negatively associated with MCS across all ethnic groups. The interaction slope for the Indians was significantly steeper compared with the Chinese and the Malays (Figure 6).

## Discussion

This article describes the pattern of HRQoL and the determinants of HRQoL in a multiethnic population cohort in Singapore. Our findings confirmed that HRQoL differed between ethnic groups replicating results of an earlier yet smaller study [17]. Physical HRQoL was higher among Chinese respondents. Malay participants on the other hand reported higher mental HRQoL despite lower socioeconomic resources and worse clinical profile than Chinese with respects to obesity, metabolic syndrome and hs-CRP. The observed ethnic differences in MCS persisted even after extensive adjustment of other sociodemographic, clinical and laboratory variables which suggests that these factors do not fully mediate the relationship between ethnicity and MCS.

The second study question addresses the contribution of sociodemographic and clinical factors on HRQoL for the total sample and across ethnic groups. The regression models for MCS and PCS explained a very modest proportion in HRQoL. Multivariate modelling indicated worse clinical profile and poor socioeconomic status were associated with lower PCS and MCS in line with previous work [17]. The link between socioeconomic standings and health outcome is undisputed [39]; the study findings showed that similar associations are also evident for HRQoL [40], [41]. Financial resources are intimately linked to environmental and living conditions and may also impact upon health care utilization and medical decision making especially in settings like Singapore where there is no free health coverage since healthcare and treatment are based on a fee for service system. Although Singapore may have less poverty-stricken people compared to other South East Asian countries, those who come from lower income families could be predisposed to more health-risk environments and may be less likely to utilize health care services adequately and/or afford optimally treatment prescribed after point of contact.

Notably, the effects of age were only observed for MCS in line with previous studies reporting higher life satisfaction amongst elderly [42]. The relationship of age with emotional well-being has often been described as ‘non-linear’ (with "middle age" respondents often having relatively low mental health scores and “older age” having higher or better emotional well-being). Older persons appear to use different reference points to evaluate their HRQoL than do younger persons. Older respondents tend to downplay the negative aspects of situations giving them neutral meaning compared to younger adults [43]. Also, changes in expectations about health and well-being across the lifespan may explain the association between age and emotional well-being in our study cohort [44]. Old age can be seen as a period of life in which one is free to explore personal fulfilment, self realization and leisure as there is more autonomy from structured roles (e.g. parenthood; employment) [45]. The lack of significant associations with PCS and age is also somewhat unexpected as advancing age brings about physical deconditioning, higher incidence of chronic illness and functional dependency. This pattern of results suggests that caution is warranted in assuming that HRQoL impairments would inevitably accompany older age.

Study findings on inter-ethnic variation on the effects of certain sociodemographic and clinical factors on HRQoL are novel and compelling as they suggest non-uniform effects across different groups. Although there are previous findings on differences in HRQoL determinants between European, Latino, African American and Asian American patients [46], [47], this is the first study to document an even more ‘fine’ ethnic variation among three Asian ethnic groups: Chinese, Indian and Malay. Using comprehensive multivariate modeling to test both main and interaction effects, we were able to show that the effect of specific factors within each cluster of variables varied as function of ethnicity.

Gender effects were more pronounced for Malay and Indian respondents. Although the main effect of gender was noted too in that females fared worse than male in physical HRQoL, the interaction effects indicated that effects were not uniform. Female gender in Malays and Indians was associated with poorer PCS, replicating previous community-based and patient research across different settings [41]–[47]. The likely explanations for the gender effects on HRQoL remain speculative but are thought to involve a complex interplay of biological, psychosocial and lifestyle factors/issues. What is not clear however is the non-uniformity in gender effects across ethnic groups. Future work should explore whether any potential differential social roles or expectations for female among ethnic groups may differentially affect HRQoL by perhaps facilitating or hindering adoption of healthy lifestyle and physical wellness.

The effects of certain comorbid conditions were also variable across ethnic group. Malay participants were the least adversely affected by stroke compared with Chinese or Indians. Although it is possible that the generally larger family units among Malays may play a positive role in alleviating the negative effects of stroke, this did not appear to be well accounted by the family functioning measure (FFM). Perhaps FFM does not capture every critical aspect of the degree of family support that would expectedly be rendered by family members in the event of a stroke for the Malays. Musculoskeletal conditions are the next comorbidity that caused significant deterioration of PCS among the three ethnic groups. Like stroke, many musculoskeletal disorders exert a profound negative impact on activities of daily living and lifestyle such as mobility and ability to cope with various occupations due to limitations by pain [48], [49]. In both musculoskeletal conditions and gender, Chinese were least affected, followed by the Malays and the Indians in descending order. The reasons for these ethnic differences at present remain intriguing and worthy of further research.

Among the list of comorbidities evaluated in this study, cancer was initially significant when it was selected as the main effect in PCS but lost its significance when interaction of stroke by ethnicity and musculoskeletal conditions by ethnicity were forward selected despite cancer being the top killer disease in Singapore. This could possibly be due to stroke and musculoskeletal disorders being much more crippling and debilitating than cancer. Also, certain cancers are now increasingly more curable or controllable and associated with longer survivals with correspondingly better quality of life than previously. Notably, hypertension is also not a significant predictor of either PCS or MCS which well supports its notorious reputation of being a ‘silent killer’ due to its largely asymptomatic condition till major target organ damage begin to manifest.

For MCS, fasting serum insulin turned up to be a negative predictor across all ethnic groups. It was interesting to find that the Indians were most adversely affected by insulin levels compared with either the Malays or the Chinese. Serum insulin usually reflects the severity of insulin resistance and corresponds to the degree of obesity. As such, given that the Indians belong to the ethnic group with the highest prevalence of diabetes mellitus in this country, this may possibly explain why Indians are susceptible to greater declines in MCS. It remains uncertain if insulin has a direct influence on cognitive and behavioral functions to explain this phenomenon. In general however, there was an apparent lack of significant multivariate associations between HRQoL and most laboratory assays/measures of various blood analytes. Although indicative of poor health or increased health risk, most laboratory abnormalities are asymptomatic until gross clinical dysfunction occurs which may explain the lack of measurable effect on individuals’ rating of HRQoL. It is finally important to note that the amount of variability in HRQoL explained by demographic, comorbidity and other clinical/biochemical markers in all models is rather small. This means that HRQoL ideally needs to be assessed by self-report rather than inferred from laboratory data or simply by comorbidity. It also suggests that other factors not measured in this study, i.e. perceived symptoms, life events, mood, social support/integration, coping skills or attitudes/expectations related to health, life in general and/or spirituality/religiosity may be more important contributors of HRQoL [46], [50]. These should be considered in future research.

Perception of better family interactions was associated with higher mental HRQoL in all groups. The effect of family functioning with MCS was not dependent on marital status. Although married respondents reported higher family functioning compared to non-married respondents, the effect of better family interaction was also found to be beneficial in respondents that were non-married, indicating that both immediate and extended family relationships/networks may be contributing to better mental HRQoL. This may be particularly the case in Asian cultures where values of interdependence emphasize the importance of family relationships and well-being [51]. Highly satisfying family relationships are shown to be more important than relationship status, of which the association to HRQoL was mixed. Marital status was associated with better physical HRQoL in the total sample relative to widowhood in agreement with previous evidence [52], [53] yet had no association with mental HRQoL. This may be because the necessary level of detail of individuals’ circumstances is unavailable. The influence of marriage on emotional well being may depend on the circumstances of the relationship. Some studies have shown that the quality of marriage rather than marriage itself is the most important influence upon emotional well-being [54].

### Strengths and Limitations

This study has several strengths, including the use of nationally representative data, stratified randomized recruitment and large sample size to allow us to perform ethnic subgroups analyses, and the assessment and inclusion of a range of demographic, socioeconomic and clinical/biochemical and laboratory parameters in the analyses. But it is also limited by the use of cross-sectional data, the use of self-report comorbidities, which albeit commonly used in epidemiological studies and generally considered to be reasonably accurate [55] may be influenced by recall and interpretation biases. Lastly there was a considerable amount of missing data with respect to income and some lab measures that reduced our sample size to more than half of original cohort. It is important however to note that complete data sample was still large to ensure a sufficiently powered study and the sensitivity analyses performed without the inclusion of income replicated the patterns of results, both of which provided assurance that the observed associations are robust. Further prospective research is needed to understand the relationship of these factors over time with HRQoL across ethnic groups, the drivers for any ethnic variation and to expand the focus to other variables, namely life experience, attitudes or expectations that may be driving ethnic differences in HRQoL.

## Supporting Information

Table S1 **Posthoc analysis**
(XLSX)

Table S2 **Univariate analysis**
(XLSX)

Table S3 **PCS hierarchical regression model (from Model 1A to 5B)**
(XLS)

Table S4 **MCS hierarchical regression model (from Model 1A to 5B)**
(XLS)

## References

[1] (2011) Singapore Department of Statistics, key demographic indicators, 1970–2011. http://www.singstat.gov.sg/stats/themes/people/popnindicators.pdf.

[2] Cella D, Yount S, Rothrock N, Gershon R, Cook K, et al (2007) The Patient-Reported Outcomes Measurement Information System (PROMIS): progress of an NIH Roadmap cooperative group during its first two years. Med Care 45: S3–S11. 10.1097/01.mlr.0000258615.42478.55 PMC2829758 17443116

[3] Dawson J, Doll H, Fitzpatrick R, Jenkinson C, Carr AJ (2010) The routine use of patient reported outcome measures in healthcare settings. BMJ 340: c186. 20083546 10.1136/bmj.c186

[4] Greenhalgh J (2009) The applications of PROs in clinical practice: what are they, do they work, and why? Qual Life Res 18: 115–123. 19105048 10.1007/s11136-008-9430-6

[5] (2009) Guidance on the routine collection of Patient Reported Outcome Measures (PROMs). http://www.dh.gov.uk/.

[6] (2012) PROMIS: Dynamic tools to measure health outcomes from the patient perspective. http://www.nihpromis.org/.

[7] Revicki DA, Osoba D, Fairclough D, Barofsky I, Berzon R, et al (2000) Recommendations on health-related quality of life research to support labeling and promotional claims in the United States. Qual Life Res 9: 887–900. 11284208 10.1023/a:1008996223999

[8] Dixon D, Pollard B, Johnston M (2007) What does the chronic pain grade questionnaire measure? Pain 130: 249–253. 17257751 10.1016/j.pain.2006.12.004

[9] Unruh M, Miskulin D, Yan G, Hays RD, Benz R, et al (2004) Racial differences in health-related quality of life among hemodialysis patients. Kidney Int 65: 1482–1491. 15086492 10.1111/j.1523-1755.2004.00529.x

[10] Idler EL, Benyamini Y (1997) Self-rated health and mortality: a review of twenty-seven community studies. J Health Soc Behav 38: 21–37. 9097506

[11] Lim WY, Ma S, Heng D, Bhalla V, Chew SK (2007) Gender, ethnicity, health behaviour & self-rated health in Singapore. BMC Public Health 7: 184. 17655774 10.1186/1471-2458-7-184 PMC1976324

[12] Quinten C, Coens C, Mauer M, Comte S, Sprangers MA, et al (2009) Baseline quality of life as a prognostic indicator of survival: a meta-analysis of individual patient data from EORTC clinical trials. Lancet Oncol 10: 865–871. 19695956 10.1016/S1470-2045(09)70200-1

[13] Doll HA, Petersen SE, Stewart-Brown SL (2000) Obesity and physical and emotional well-being: associations between body mass index, chronic illness, and the physical and mental components of the SF-36 questionnaire. Obes Res 8: 160–170. 10757202 10.1038/oby.2000.17

[14] Wee HL, Wu Y, Thumboo J, Lee J, Tai ES (2010) Association of body mass index with Short-Form 36 physical and mental component summary scores in a multiethnic Asian population. Int J Obes (Lond) 34: 1034–1043. 20142824 10.1038/ijo.2010.24

[15] Otten MW Jr, Teutsch SM, Williamson DF, Marks JS (1990) The effect of known risk factors on the excess mortality of black adults in the United States. JAMA 263: 845–850. 2296146

[16] Ng TP, Lim LC, Jin A, Shinfuku N (2005) Ethnic differences in quality of life in adolescents among Chinese, Malay and Indians in Singapore. Qual Life Res 14: 1755–1768. 16119186 10.1007/s11136-005-1741-2

[17] Thumboo J, Fong KY, Machin D, Chan SP, Soh CH, et al (2003) Quality of life in an urban Asian population: the impact of ethnicity and socio-economic status. Soc Sci Med 56: 1761–1772. 12639592 10.1016/s0277-9536(02)00171-5

[18] Wee HL, Li SC, Cheung YB, Fong KY, Thumboo J (2006) The influence of ethnicity on health-related quality of life in diabetes mellitus: a population-based, multiethnic study. J Diabetes Complications 20: 170–178. 16632237 10.1016/j.jdiacomp.2005.06.010

[19] Dalan R, Jong M, Chan SP, Hawkins R, Choo R, et al (2010) High-sensitivity C-reactive protein concentrations among patients with and without diabetes in a multiethnic population of Singapore: CREDENCE Study. Diabetes Metab Syndr Obes 3: 187–195. 21437088 PMC3047995

[20] Khoo CM, Liew CF, Chew SK, Tai ES (2007) The impact of central obesity as a prerequisite for the diagnosis of metabolic syndrome. Obesity (Silver Spring) 15: 262–269. 17228055 10.1038/oby.2007.559

[21] Liew CF, Seah ES, Yeo KP, Lee KO, Wise SD (2003) Lean, nondiabetic Asian Indians have decreased insulin sensitivity and insulin clearance, and raised leptin compared to Caucasians and Chinese subjects. Int J Obes Relat Metab Disord 27: 784–789. 12821962 10.1038/sj.ijo.0802307

[22] Tan CE, Ma S, Wai D, Chew SK, Tai ES (2004) Can we apply the National Cholesterol Education Program Adult Treatment Panel definition of the metabolic syndrome to Asians? Diabetes Care 27: 1182–1186. 15111542 10.2337/diacare.27.5.1182

[23] Bhalla V, Fong CW, Chew SK, Satku K (2006) Changes in the levels of major cardiovascular risk factors in the multi-ethnic population in Singapore after 12 years of a national non-communicable disease intervention programme. Singapore Med J 47: 841–850. 16990958

[24] (2001) Singapore Census of Population; Cheung P, editor: Singapore Department of Statistics.

[25] (2007) National Health Surveillance Survey 2007. http://www.moh.gov.sg/mohcorp/uploadedFiles/Publications/Reports/2009/nhss2007.pdf.

[26] Bassett DR Jr (2003) International physical activity questionnaire: 12-country reliability and validity. Med Sci Sports Exerc 35: 1396. 12900695 10.1249/01.MSS.0000078923.96621.1D

[27] Craig CL, Marshall AL, Sjostrom M, Bauman AE, Booth ML, et al (2003) International physical activity questionnaire: 12-country reliability and validity. Med Sci Sports Exerc 35: 1381–1395. 12900694 10.1249/01.MSS.0000078924.61453.FB

[28] Ware JE, Jr., Kosinski M, Keller SD (1994) SF-36. physical and mental health summary scales: A user’s manual. Boston: The Health Institute.

[29] Ware JE, Jr., Snow KK, Kosinski M, Gandek B (1993) SF-36 health survey: Manual and interpretation guide. Boston: The Health Institute.

[30] Chang YW, Chen WL, Lin FG, Fang WH, Yen MY, et al. Frailty and its impact on health-related quality of life: a cross-sectional study on elder community-dwelling preventive health service users. PLoS One 7: e38079. 22662268 10.1371/journal.pone.0038079 PMC3360631

[31] Maruish M (2007) User’s Manual for the SF-36v2 Health Survey. Lincoln. R.I. : QualityMetric Incorporated.

[32] Ramachandran V, Malaisamy M, Ponnaiah M, Kaliaperuaml K, Vadivoo S, et al. Impact of chikungunya on health related quality of life chennai, South India. PLoS One 7: e51519. 10.1371/journal.pone.0051519 PMC3520806 23251562

[33] Vathesatogkit P, Sritara P, Kimman M, Hengprasith B, T ES, et al. Associations of lifestyle factors, disease history and awareness with health-related quality of life in a Thai population. PLoS One 7: e49921. 23189172 10.1371/journal.pone.0049921 PMC3506606

[34] Ware JE Jr, Gandek B (1998) Overview of the SF-36 Health Survey and the International Quality of Life Assessment (IQOLA) Project. J Clin Epidemiol 51: 903–912. 9817107 10.1016/s0895-4356(98)00081-x

[35] Ware JE Jr, Kosinski M, Gandek B, Aaronson NK, Apolone G, et al (1998) The factor structure of the SF-36 Health Survey in 10 countries: results from the IQOLA Project. International Quality of Life Assessment. J Clin Epidemiol 51: 1159–1165. 9817133 10.1016/s0895-4356(98)00107-3

[36] Sherbourne CD, Kamberg CJ (1992) Measuring functioning and well-being: The medical outcomes study approach. In: Stewart AL, Ware JE, Jr., editors. Social functioning: Family and martial functioning measures. Dueham: Duke University Press. 193–193.

[37] Thumboo J, Fong KY, Chan SP, Leong KH, Feng PH, et al (1999) Validation of the medical outcomes study family and marital functioning measures in SLE patients in Singapore. Lupus 8: 514–520. 10483028 10.1191/096120399678840747

[38] Matthews DR, Hosker JP, Rudenski AS, Naylor BA, Treacher DF, et al (1985) Homeostasis model assessment: insulin resistance and beta-cell function from fasting plasma glucose and insulin concentrations in man. Diabetologia 28: 412–419. 3899825 10.1007/BF00280883

[39] Adler NE, Boyce T, Chesney MA, Cohen S, Folkman S, et al (1994) Socioeconomic status and health. The challenge of the gradient. Am Psychol 49: 15–24. 8122813 10.1037//0003-066x.49.1.15

[40] Pappa E, Kontodimopoulos N, Papadopoulos AA, Niakas D (2009) Assessing the socio-economic and demographic impact on health-related quality of life: evidence from Greece. Int J Public Health 54: 241–249. 19424661 10.1007/s00038-009-8057-x

[41] Pekmezovic T, Popovic A, Tepavcevic DK, Gazibara T, Paunic M (2010) Factors associated with health-related quality of life among Belgrade University students. Qual Life Res 20: 391–397. 20878242 10.1007/s11136-010-9754-x

[42] Netuveli G, Wiggins RD, Hildon Z, Montgomery SM, Blane D (2005) Functional limitation in long standing illness and quality of life: evidence from a national survey. BMJ 331: 1382–1383. 16339249 10.1136/bmj.331.7529.1382 PMC1309649

[43] Diehl M, Coyle N, Labouvie-Vief G (1996) Age and sex differences in strategies of coping and defense across the life span. Psychol Aging 11: 127–139. 8726378 10.1037//0882-7974.11.1.127

[44] Kutner NG (1994) Psychosocial issues in end-stage renal disease: aging. Adv Ren Replace Ther 1: 210–218. 7614324 10.1016/s1073-4449(12)80003-3

[45] Bowling A (2005) Ageing well: quality of life in old age. Maidenhead: Open University Press.

[46] Ashing-Giwa KT, Lim JW Predicting physical quality of life among a multiethnic sample of breast cancer survivors. Qual Life Res 19: 789–802. 20340047 10.1007/s11136-010-9642-4

[47] Giedzinska AS, Meyerowitz BE, Ganz PA, Rowland JH (2004) Health-related quality of life in a multiethnic sample of breast cancer survivors. Ann Behav Med 28: 39–51. 15249258 10.1207/s15324796abm2801_6

[48] Hudson M, Thombs BD, Steele R, Panopalis P, Newton E, et al (2009) Health-related quality of life in systemic sclerosis: a systematic review. Arthritis Rheum 61: 1112–1120. 19644906 10.1002/art.24676

[49] Lee DM, Weinblatt ME (2001) Rheumatoid arthritis. Lancet 358: 903–911. 11567728 10.1016/S0140-6736(01)06075-5

[50] Meyerowitz BE, Richardson J, Hudson S, Leedham B (1998) Ethnicity and cancer outcomes: behavioral and psychosocial considerations. Psychol Bull 123: 47–70. 9461853 10.1037/0033-2909.123.1.47

[51] Markus HR, Kitayama S (1991) Culture and the self: Implications for cognition, emotion, and motivation. Psychol Rev 98: 224–253.

[52] Mineau GP, Smith KR, Bean LL (2002) Historical trends of survival among widows and widowers. Soc Sci Med 54: 245–254. 11824929 10.1016/s0277-9536(01)00024-7

[53] Wilcox S, Evenson KR, Aragaki A, Wassertheil-Smoller S, Mouton CP, et al (2003) The effects of widowhood on physical and mental health, health behaviors, and health outcomes: The Women's Health Initiative. Health Psychol 22: 513–522. 14570535 10.1037/0278-6133.22.5.513

[54] Gove WR, Hughes M, Style CB (1983) Does marriage have positive effects on the psychological well-being of the individual? J Health Soc Behav 24: 122–131. 6886367

[55] Katz JN, Chang LC, Sangha O, Fossel AH, Bates DW (1996) Can comorbidity be measured by questionnaire rather than medical record review? Med Care 34: 73–84. 8551813 10.1097/00005650-199601000-00006

